# Ultrasonographic assessment of ossification of the distal radial epiphysis for estimating forensic age

**DOI:** 10.1007/s00414-021-02521-2

**Published:** 2021-02-20

**Authors:** Oguzhan Ekizoglu, Ali Er, Asli Dilara Buyuktoka, Mustafa Bozdag, Gokce Karaman, Negahnaz Moghaddam, Silke Grabherr

**Affiliations:** 1 Department of Forensic Medicine, Tepecik Training and Research Hospital, Izmir, Turkey; 2 Unit of Forensic Imaging and Anthropology, University Center of Legal Medicine, Lausanne-Geneva, Switzerland; 3 Department of Radiology, Tepecik Training and Research Hospital, Izmir, Turkey; 4 Ministry of Justice, Council of Forensic Medicine, Manisa, Turkey; 5 Swiss Human Institute of Forensic Taphonomy, University Center of Legal Medicine, Lausanne-Geneva, Switzerland; 6 University Center of Legal Medicine, Lausanne-Geneva, Switzerland

**Keywords:** Age estimation, Distal radial epiphysis, Ultrasonography, Minors

## Abstract

Since forensic age estimation is not a valid medical indication, research on the use of nonionizing methods is increasing. Ultrasonography is a radiological approach that protects patients from radiation exposure and offers special convenience to them. In this study, ultrasonography was used for age estimation by investigating the degree of ossification of the distal radial epiphysis. Its applicability on the Turkish population was investigated. The left wrist of 688 (322 males, 366 females) patients between the ages of 9 and 25 years was prospectively evaluated by ultrasonography. The intra- and interobserver reliabilities in evaluating the distal radial epiphysis and Cohen’s kappa statistics show that the interobserver error was very low, and the kappa value was found to be 0.919. Stage 3 and 4 ossification of the distal radial epiphysis was first detected at age 14.3 and 15.3 years in males and 12.7 and 14.8 years in females, respectively. The data obtained may help determine legally critical age limits of 14 and 15. Although it does not seem useful for the age of 18, ultrasonography may be recommended in selected cases as a fast, inexpensive, frequently reproducible radiological method without concern about radiation and without a predictable health risk.

## Introduction

Due to the increase in cross-border migration in recent years, the presence of individuals with unconfirmed birth date has increased in many countries, especially in Europe. As a result, age estimation has become a frequently used forensic medical evaluation under civil and criminal law. As individuals under the age of 18 have different rights than adults within the scope of asylum in Europe [1–4], forensic age estimation may be required to assess the status of adolescents seeking asylum. On the other hand, to determine which of the criminal laws applicable to adults or children has to be applied, forensic age estimation is mandatory for individuals whose birth date has not been confirmed [3]. In criminal cases, the important age limits in terms of determining criminal liability are 14, 18, and 21 years in many countries [3, 4]. Another area where age estimation gains importance is in sports competitions. Indeed, ensuring that athletes participate in sports competitions in appropriate age groups is important in terms of ensuring fair competition and protecting the health of athletes [5, 6].

The maturity state of the hand and wrist skeleton is one of the main investigated areas, showing the development of the skeletal system [7–10]. The hand skeleton has special importance in age estimation up to approximately 18 years of age, when the developmental process ends [7]. The degree of maturation of the distal radial epiphysis is also a major evaluation in the area of forensic age estimation. As the majority of people are right-hand dominant, traumas to the right hand and wrist are more likely, and skeletal development may be affected due to continuous use; thus, it has been stated that X-ray images taken for the left hand should be used in forensic age estimation [11].

Noninvasive age estimation methods have been recommended due to consideration of ethical concerns in minors [2, 12]. An increasing number of published studies, especially for nonionizing-based methods, and experimental methods [13] have been conducted in recent years. Previous studies have reported that ultrasonography (USG) as a nonionizing method is an easily accessible, low-cost, tolerable, and alternative method for age estimation [14–17]. Past studies using USG for forensic age estimation have examined the iliac crest [18, 19], elbow [20], hand-wrist [21–25], clavicle [26–28], knee [29], greater trochanter [30], and distal fibula [31].

The aim of this study was to ultrasonographically evaluate ossification of the distal radius epiphysis to show its utility in forensic age estimation in living individuals. We wanted to assess the usability of USG, as a nonionizing method, for pediatric age groups. This study also contributes to the validation of the methodology of Schmidt et al. [25] and compares the result obtained by those authors to our Turkish population.

## Materials and methods

### Study design and subjects

This study was planned prospectively and carried out on volunteers. After the evaluation of the patients who applied to the local radiology clinic by requesting USG for different clinical indications, they and their legal guardians were informed about the study. A oral and written consent was obtained from those who agreed to participate in this study.

The study was approved by the local ethics committee. No evaluation was made regarding the socioeconomic level and ethnicity information of the individuals included in the study. Exclusion criteria are any pathology of the hand-wrist (e.g., tumor, fracture, infection, surgical fixation), patients with neoplastic disorders, and patients undergoing radiotherapy or chemotherapy. Medical anamnesis for all cases was performed in terms of pathologies that may affect skeletal development, and past medical records were examined. Fourteen cases were not included in the study because they were followed up in pediatric oncology. A total of 366 females and 322 males aged between 9 and 25 years were included. The distribution of the cases by age and sex is shown in Table 1.

**Table 1:** Age distribution of female and male cases

Age (years)	Female (*N*)	Male (*N*)	Total (*N*)
9	1	1	2
10	25	12	37
11	17	29	46
12	14	21	35
13	27	28	55
14	35	41	76
15	48	35	83
16	32	29	61
17	46	30	76
18	36	18	54
19	22	12	34
20	15	16	31
21	10	18	28
22	13	16	29
23	14	10	24
24	10	4	14
25	1	2	3
Total	366	322	688

*N* number of cases

### USG examination

B-mode ultrasound was performed distal to the left radius of the patients. The Toshiba Aplio 300 system (Toshiba Medical Systems, Tokyo, Japan), including software with a combined autocorrelation and a multifrequency linear probe (frequency 10 MHz and frequency range 7.0–12.0 MHz), was used.

USG examinations were performed by two observers, each one examining the patients alone. Both observers were radiologists with 10 years of experience in forensic age estimation for observer 1 and 2 years for observer 2. During the exams, the patients were sitting on the patient table to facilitate evaluation of the left radius. For USG examination of the radius, the palmar face of the forearm-hand was positioned superior. After the probe was placed parallel to the long axis of the radius, the evaluation was made by moving it lateral to medial and then medial to lateral. The chronological age of the patients was not known to the observers.

Although it would have been interesting to ask each observer to examine each subject independently, in order to be able to compare their estimations, this was not possible in this study as it would lead to prolonged evaluations for the patients. However, in order to have an idea about the interobserver agreement of the method, we were able to double the examination in 50 cases. In those cases, the subjects were first examined by observer 1, than by observer 2.

As shown in Table 2, the staging system described by Schulz et al. [27] for the clavicle, modified by Schmidt et al. [25] for the distal radius, was used.

**Table 2:**
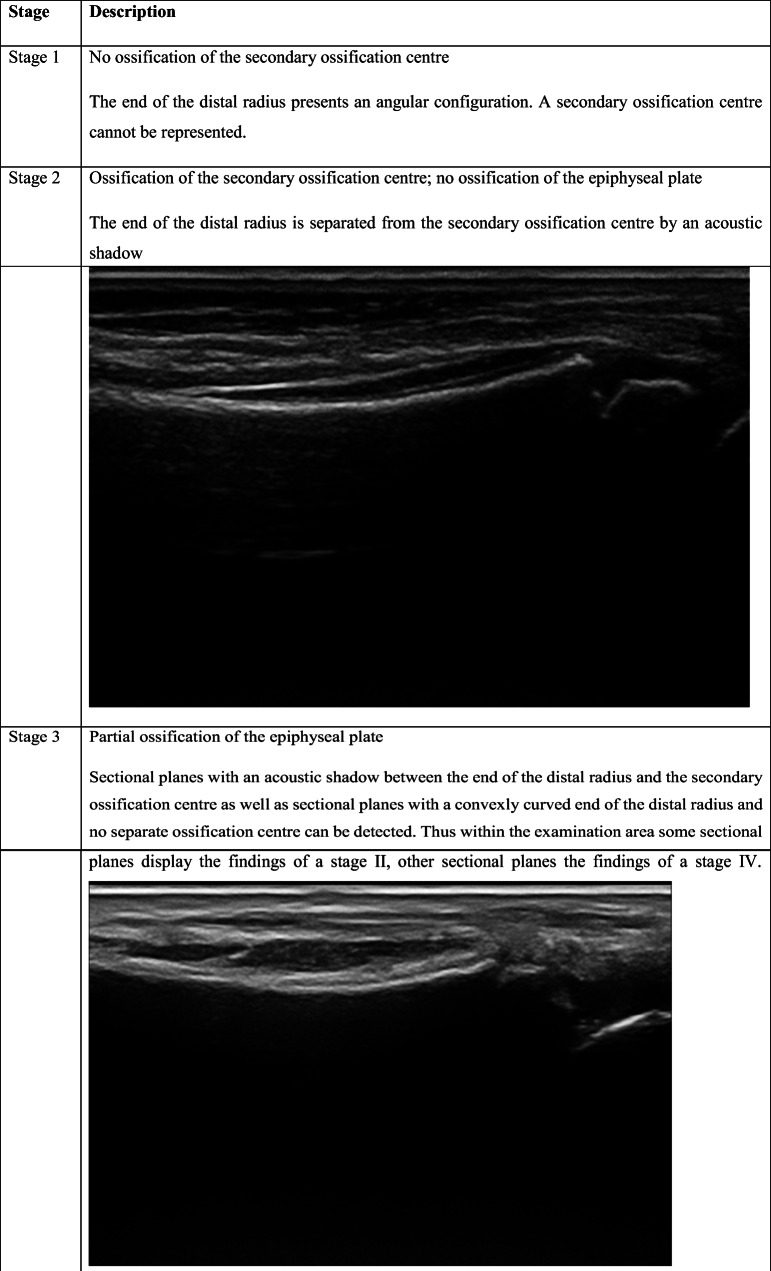

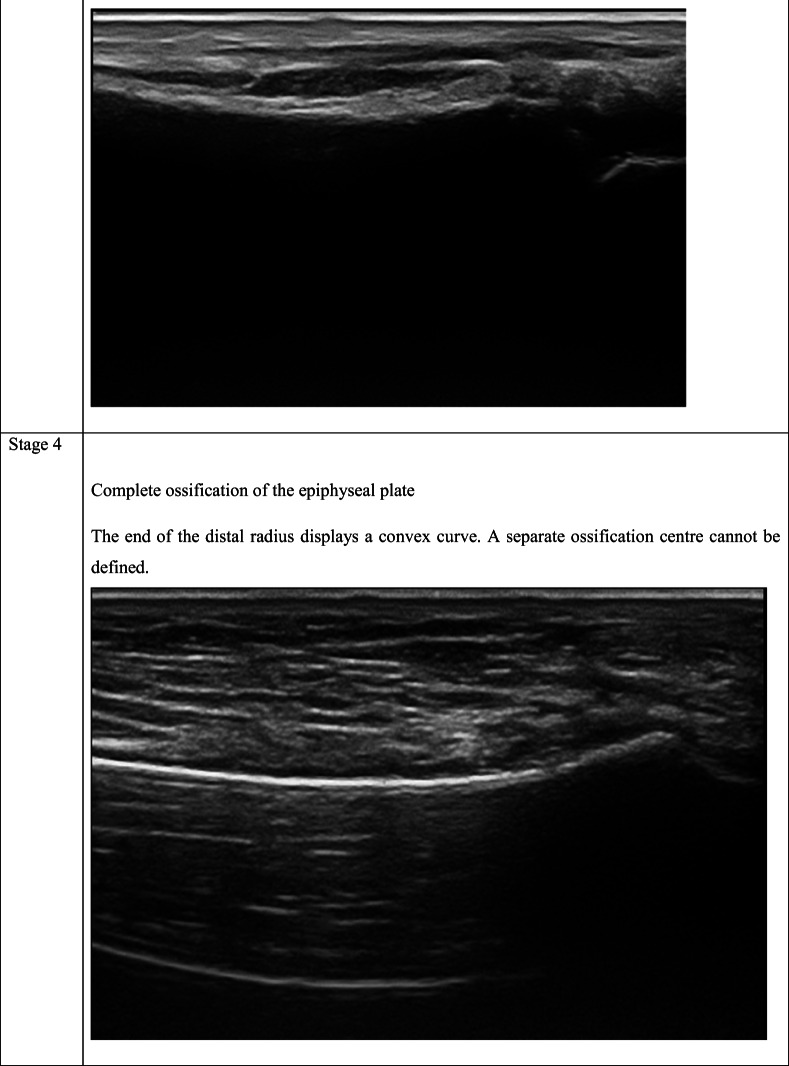
The ultrasonography staging system described by Schulz et al. [27] for the clavicle modified by Schmidt et al. [25] for the distal radius. Ultrasonographic images for each stage were shown in the table

### Statistical analysis

Statistical Package for the Social Sciences (SPSS) software (ver. 17; IBM Corporation, Armonk, NY, USA) was used for the statistical analyses. Data are expressed as means or medians, with standard deviations (SDs), lower and upper quartiles, and minimum and maximum values, as appropriate. Associations between age and ossification stage were evaluated using Spearman’s correlation analysis. Between-sex comparisons were carried out using the Mann–Whitney *U* test. A *p* value < 0.01 was taken to reflect statistical significance.

The extent of interobserver agreement was evaluated using Cohen’s test, and the weighted κ value and agreement ratio were calculated. The values were determined according to the system developed by Altman [32].

## Results

A total of 688 patients were included in this study (322 male and 366 female patients; age range, 9–25 years). The mean ages of the male and female patients were 16.30 ± 3.68 years and 16.59 ± 3.63 years, respectively. Interobserver evaluation indicated good reproducibility and consistency of the method. For interobserver reliability, Cohen’s kappa test value was found to be 0.919, indicating excellent agreement.

Stage 1 was not observed in any of the patients included in the study.

The first ages detected for males were 9.4 years in stage 2, 14.3 years in stage 3, and 15.3 years in stage 4. In females, the first detected ages were 9.9 years, 12.7 years, and 14.8 years for stages 2, 3, and 4, respectively.

Table 3 shows the mean age for each stage in both sexes, as well as the standard deviations, minimum and maximum ages, medians, and lower and upper quartiles.

**Table 3:** Minimum and maximum ages with means ± SDs. Lower and upper quartiles and medians at all stages of distal radial epiphysis

Stage	Sex	*N*	Mean ± SD	Min-Max	LQ; UQ; Median
Stage 2	Female	95	12.43 ± 1.80	9.9–17.5	10.70; 14.00; 12.30
	Male	122	12.83 ± 1.60	9.4–16.8	11.37; 14.00; 12.75
Stage 3	Female	110	16.11 ± 1.86	12.7–21.8	14.75; 17.30; 15.80
	Male	92	16.43 ± 1.81	14.3–22.8	14.80; 17.40; 16.05
Stage 4	Female	161	19.38 ±2.76	14.8–25.3	17.30; 21.60; 18.90
	Male	108	20.12 ± 2.55	15.3–25.1	18.20;22.10; 20.15

*N* number of cases, *SD* standard deviation, *Min* minimum age, *Max* maximum age, *LQ* lower quartiles, *UQ* upper quartiles

In this study, Spearman’s rank correlation analysis indicated a significant positive relationship between age and ossification stage of the distal radial epiphysis (total group: rho = 0.835, *p* < 0.001; male: rho = 0.864, *p* < 0.001; female: rho = 0.803, *p* < 0.001). In comparisons between sexes, the data revealed statistically significant differences for stage 4 (*p* < 0.021). No statistically significant difference was found for the other stages.

## Discussion

Nonionizing methods available to estimate age in minors are crucial. In this study, a published method defined by Schmidt et al. [25] was used on a large sample of the Turkish population. In this approach, ossification of the distal radius epiphysis was assessed by USG, and the results were discussed according to the minimal age concept. For this purpose, four stages were used, but three stages were established, similar to the original study. The results show that the minimal ages of patients at stage 3 and stage 4 were 12.7 and 14.8 years for females, and 14.3 and 15.3 years for males, respectively.

USG requires a high level of experience and is associated with less objectivity than other radiological methods. The reliability of the results depends significantly on the experience level of each observer [14–17, 22]. Indeed, the exact evaluation is important in forensic dating cases, considering the judicial-civil implications of the result. Despite these concerns, interobserver agreement was found to be high in past forensic age estimation studies. Moreover, interobserver agreement (weighted kappa coefficients) was reported to be 0.95 for the distal femoral epiphysis and 0.81 for the proximal tibial epiphysis [29], 0.898 for the distal radius epiphysis [25], 0.946 for the iliac crest [18], and 0.969 for the clavicle [28]. Although it was not possible in our study to examine each subject by all observers, we were able to have examinations performed by two different observers in 50 cases. Therefore, in this limited population, we could evaluate the interobserver agreement, which showed a weighted kappa coefficient of 0.919 and therefore an excellent agreement.

It is noteworthy that there are limited studies on the evaluation of wrist skeletal maturity using ultrasound for forensic age estimation [24, 25].

Schmidt et al. evaluated the distal radial epiphysis using the USG staging system in 39 cases, but it was stated that this pilot study should be supported by studies involving more cases [24], and Schmidt et al. later published the results of a study evaluating 615 cases [25] in which it was stated that stage 3 female cases were older than 13.4 years old and that stage 3 male cases were older than 14.3 years [25]. In the same study, stage 4 female cases were found to be older than 15.0 years and male cases older than 15.2 years [25]. The data of the present study for males appears to be compatible with the study of Schmidt et al. [25]. The minimum ages were lower, with 12.7 in stage 3 females and 14.8 in stage 4 females. However, the difference was less than 1 year at each stage. In our study, the standard deviations for the mean ages for each stage varied between 1.8 and 2.7 in female cases and between 1.6 and 2.5 in male cases. In the study of Schmidt et al., it was observed that the standard deviations for the average ages for each stage varied between 1.8 and 2.7 for females and between 1.9 and 2.5 for males and were very similar to our study [25].

Due to the specific features of each radiological imaging method, it seems appropriate to evaluate the data provided by each method alone. Although X-ray and USG comparisons do not appear to be appropriate in this respect, the results of Schmidt et al. [33] and Baumann et al. [34], who used X-ray on the distal radius, can be considered due to the same approach being used for epiphyseal closure defined by the staging method. The minimal age limits for stage 3 and stage 4 were lower in both studies, and the 1–3 age difference was striking, though it varied by sex and stage. Schmidt et al. [25] reported that there are some possible causes for this situation with regard to stage 3. It has been stated that the first osseous structures that begin to form in the epiphyseal line are more difficult to be detected by sonography than by radiography; in addition, as ossification occurs in a centrifugal manner, additional time is required for the formation of the homogeneous convex cortical bone borders that form a cross-sectional image to make a diagnosis of stage 3 by sonography [25]. Since parts of the epiphyseal line that are not fully ossified are more evident by sonography than radiography, the minimal ages detected in stage 4 in sonographic examinations may be greater [25]. This delay in detecting ossification of the distal radial epiphysis by USG can also be seen in the results of two different magnetic resonance imaging (MRI) studies in German and Turkish populations at stage 3, at which partial ossification is defined. According to Timme et al. [35] and Er et al. [36], the minimum ages for stage 3 were 12.1–12.0 and 13.6 and 13.8 for men and women, respectively. Although it is obvious that all these comparisons should be repeated within the same cohort, a method of forensic medical evaluation that risks overestimating the age of a minor is disadvantageous. Indeed, it can result in the judgment of the minor by adult laws or of losing civil rights that are reserved for minors. Accordingly, the results of the USG and MRI comparative study for the knee by Hermann et al. [29] are important for supporting the detection of later stages by USG. In this study, the USG stage was found to be greater than the MRI stage in 85.7% and 50% of the cases for the femur in the “partially closed epiphysis” and “closed epiphysis” stages and in 33.3% and 38.5% for the tibia, respectively.

One of the most important questions when considering the differences between studies is ethnicity. Schmeling et al. [37] concluded that ethnicity does not have a significant effect on the ossification rate. On the other hand, low socioeconomic status may delay ossification, and consequently, the status of the socioeconomic sample has a significant effect on age determination [38]. The socioeconomic status of the cases in this study was not available. According to the Human Development Index (HDI), Germany is ranked 4th and Turkey 59th on the socioeconomic index [39]. Although the HDI provides basic information about the population in which the study is conducted, it is necessary to approach the data carefully, considering that socioeconomic differences cannot be excluded within the population itself. Although presented study was conducted prospectively, age distribution was unbalanced. It could be shows a potential selection bias and keep in mind especially lower and upper extremes of stages as a limitation.

Overlap is among the reported limitations of the USG method [2, 29]. Anatomical features that differ with age may prevent the evaluation of ossification depending on the area studied [2, 29]. Forensic age estimation for the wrist as well as clinically based studies are available, and it is known that the distal radial epiphysis is well defined on USG images [21, 22, 37]. According to the experience obtained in this study, the echogenic view of the secondary ossification center and the acoustic shadow formed are also observed along the bone surface of distal radius. When ossification between the distal of radius and the secondary ossification center has not occurred, there is no continuity on the bone surface. For this reason, the acoustic shadow does not appear in stage 2. When the integrity and consequently continuity of the bone cortex occurs in the areas with ossification, the acoustic shadow is continuously observed in these parts in stage 4. In addition, due to centrifugal ossification, there are areas where the continuity of the bone surface is formed and not yet formed. Therefore, it is seen that there is or does not have acoustic shadow in these areas. This imaging feature helps to discriminate stage 3 from stage 2 and 4. Ultrasonography offers the opportunity to evaluate different planes simultaneously during the examination. At the same time, it provides the opportunity to re-evaluate without time limitation areas where it is suspected to be reproducible. In addition to constituting a real-time review, the entire examination can be recorded in video format, allowing for later evaluation, owing to today’s developing PACS (picture archiving and communication system) capabilities. The images obtained within the scope of a standardized examination allow later evaluation by other experts on the subject.

One of the additional benefits that USG provides, especially in the child age group, is the ease of the application process compared to another nonionizing method, such as the MRI. Although MRI allows for detailed analysis, the main factors, such as the necessity to remain still during the application, the necessity of entering a closed area of the device, and the sounds of the device, are important challenges, especially for a child. Considering these difficulties, USG can provide a more comfortable radiological evaluation. Today, target-focused USG training can be offered, as in many specialties, such as emergency medicine, urology, and obstetric specialists. Forensic medicine specialists could carry out rapid age estimation in the future due to the availability of USG devices. Herrmann et al. [29] stated that the time required to examine the knee by USG is 2.65 ± 0.91 min, and therefore much lower than the one need for the age estimation by MRI (24.72 ± 2.72 min, without the positioning of patients).

The results of this study support that it is possible to determine distal radius ossification stages by USG. The degree of ossification determined by USG showed that females reached the age of 14.8 and males 15.3 years at stage 4. Since stage 5 cannot be revealed by USG, it is not useful for higher critical age limits. The ultrasonographic staging method described by Schmidt appears to be reproducible and consistent. Considering the observer-dependent error probability of USG, the findings should be recorded and re-evaluated by at least 2 experienced observers to increase the reliability of the forensic age estimates.
